# Exploring the Potential of Royal-Jelly-Incorporated Hydrogel Dressings as Innovative Wound Care Materials

**DOI:** 10.3390/ijms24108738

**Published:** 2023-05-14

**Authors:** Sonia Kudłacik-Kramarczyk, Marcel Krzan, Mateusz Jamroży, Alicja Przybyłowicz, Anna Drabczyk

**Affiliations:** 1 Department of Materials Engineering, Faculty of Materials Engineering and Physics, Cracow University of Technology, 37 Jana Pawła II Av., 31-864 Krakow, Poland; mateusz.jamrozy@student.pk.edu.pl (M.J.); alicja.przybylowicz@student.pk.edu.pl (A.P.); anna.drabczyk2@pk.edu.pl (A.D.); 2 Jerzy Haber Institute of Catalysis and Surface Chemistry, Polish Academy of Sciences, 8 Niezapominajek St., 30-239 Krakow, Poland

**Keywords:** multifunctional hydrogel dressings, wound healing, active substances, royal jelly, photopolymerization

## Abstract

The development of multifunctional dressing materials with beneficial properties for wound healing has become a recent focus of research. Many studies are being conducted to incorporate active substances into dressings to positively impact wound healing processes. Researchers have investigated various natural additives, including plant extracts and apiproducts such as royal jelly, to enhance the properties of dressings. In this study, polyvinylpyrrolidone (PVP)-based hydrogel dressings modified with royal jelly were developed and analyzed for their sorption ability, wettability, surface morphology, degradation, and mechanical properties. The results showed that the royal jelly and crosslinking agent content had an impact on the physicochemical properties of the hydrogels and their potential for use as innovative dressing materials. This study investigated the swelling behavior, surface morphology, and mechanical properties of hydrogel materials containing royal jelly. The majority of the tested materials showed a gradual increase in swelling ratio with time. The pH of the incubated fluids varied depending on the type of fluid used, with distilled water having the greatest decrease in pH due to the release of organic acids from the royal jelly. The hydrogel samples had a relatively homogeneous surface, and no dependence between composition and surface morphology was observed. Natural additives like royal jelly can modify the mechanical properties of hydrogels, increasing their elongation percentage while decreasing their tensile strength. These findings suggest possible future applications in various fields requiring high flexibility and elasticity.

## 1. Introduction

In recent times, a great interest in developing multifunctional dressing materials has been observed. Hence, current dressings are considered not only as materials protecting the wound from the external environment but those that show other useful properties, such as wound exudate sorption ability [1], high flexibility [2], anti-biofilm and promigratory [3], controlled drug delivery ability [4], or stimuli-responsive ability [5,6,7]. Many studies are currently being performed to incorporate dressing materials with an active substance, while particular attention is being directed to the substances which affect the wound-healing process positively [8,9,10,11,12].

Studies so far have focused both on developing the adequate composition of the hydrogel matrix as well as selecting additives showing beneficial properties in terms of vascular regeneration and cell proliferation [13,14]. Substances of natural [15,16,17] or synthetic [18,19] origin and their combinations [20,21] have been proposed as components of hydrogel matrixes. The investigated modifiers include antibiotics [22], herbal extracts [23], essential oils [24], and vitamins [25].

In recent times, the potential of apiproducts including, e.g., propolis [26] or honey [27] in the development of biomaterials has been noted. Importantly, royal jelly has also attracted the attention of scientists [28,29]. Royal jelly consists of various bioactive compounds, including proteins, lipids, flavonoids, and phenols. Hence, it was demonstrated in many studies that this material plays a significant role in the regulation of the blood glucose level, the work of the digestive system, and immunity [30]. Moreover, it was also reported that royal jelly exhibits antimicrobial [31,32] and antioxidant activity [33]. The properties of dressing materials modified with royal jelly have been verified; for example, by Siavash et al. Here, the application potential of royal jelly containing dressings as materials supporting diabetic foot ulcer treatment has been confirmed [34].

In this work, studies on a polyvinylpyrrolidone (PVP)-based hydrogel containing royal jelly and obtained via photopolymerization are described. To our knowledge, studies on materials with such composition (both the hydrogel matrix and the modifier in the form of royal jelly) and obtained using UV radiation have not been so far presented. Developed materials differed in royal jelly content and the amount of the crosslinking agent used during the synthesis. The materials were subsequently subjected to the analysis of their sorption ability in selected simulated physiological liquids, wettability and surface morphology by means of scanning electron microscopy (SEM). Furthermore, the tendency of the hydrogels to degrade in simulated physiological liquids was verified, as well as their mechanical properties, including tensile strength and percentage elongation. The main attention during the discussion over the results obtained was paid to the impact of the royal jelly and the crosslinking agent content on the physicochemical properties of hydrogels, as well as on their potential for application as innovative dressing materials.

## 2. Results and Discussion

### 2.1. Sorption Capacity of Hydrogels

**Figure 1:**
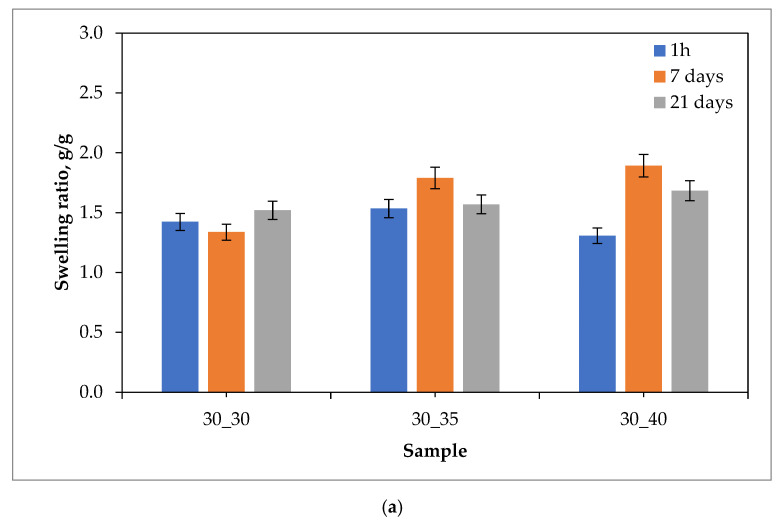

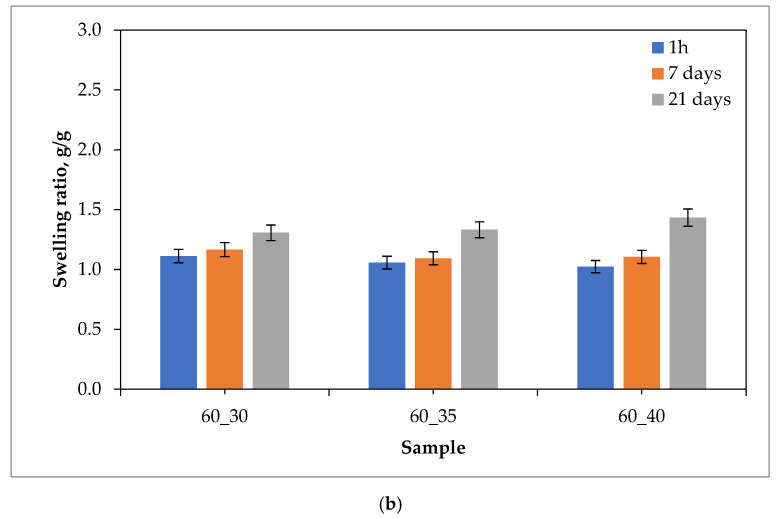
Results of swelling studies of hydrogels containing 2.0 g (**a**) and 4.0 g (**b**) of royal jelly in distilled water.

**Figure 2:**
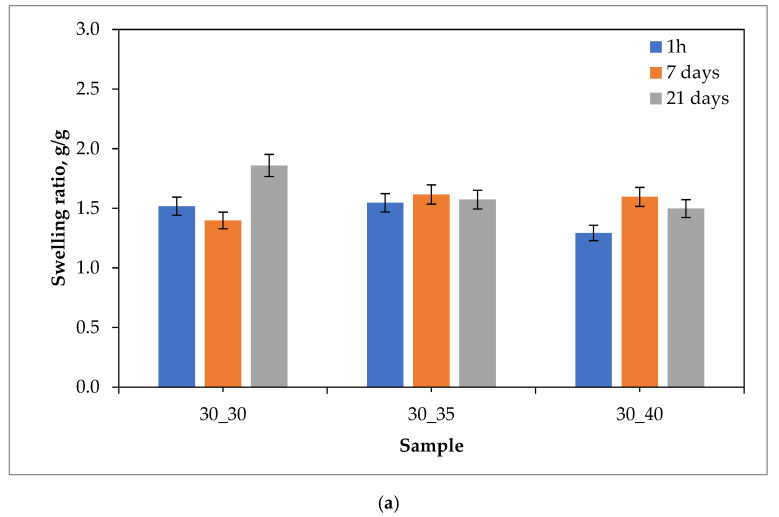

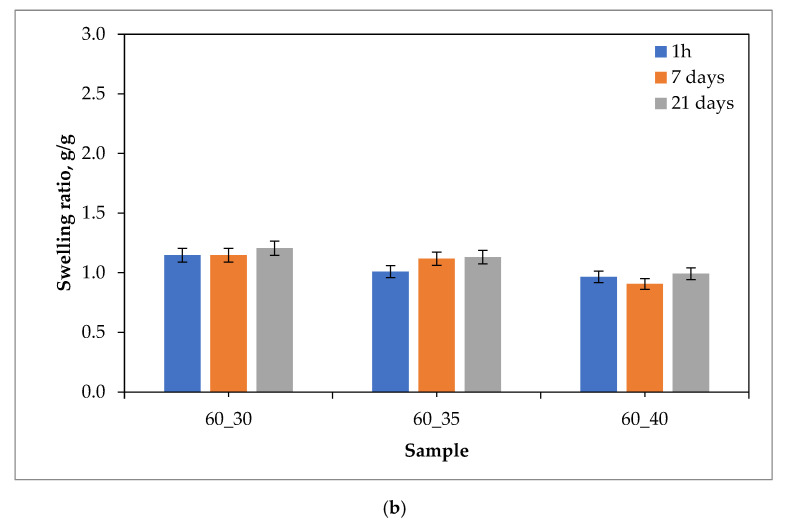
Results of swelling studies of hydrogels containing 2.0 g (**a**) and 4.0 g (**b**) of royal jelly in SBF.

**Figure 3:**
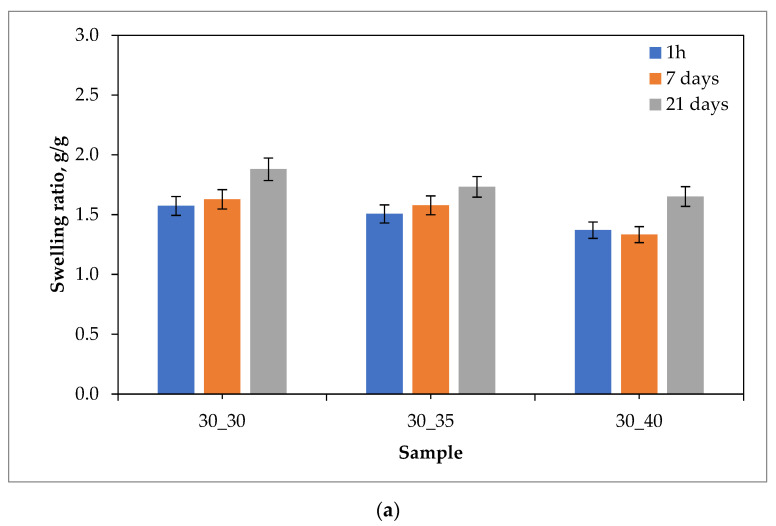

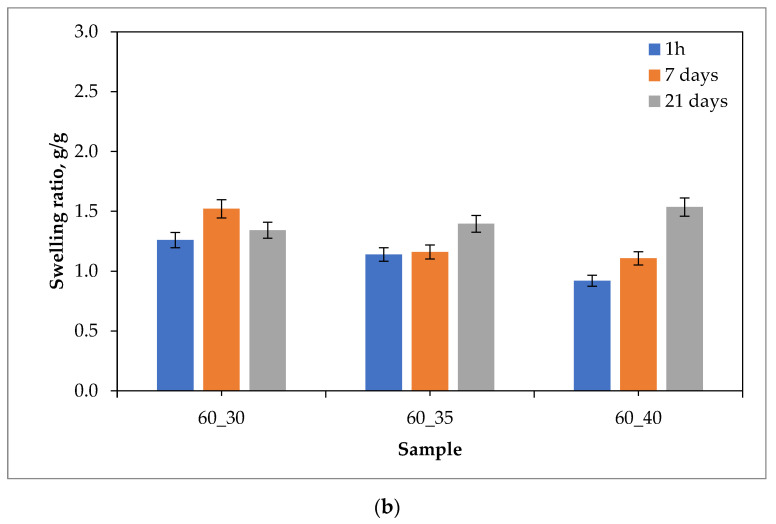
Results of swelling studies of hydrogels containing 2.0 g (**a**) and 4.0 g (**b**) of royal jelly in Ringer liquid.

**Figure 4:**
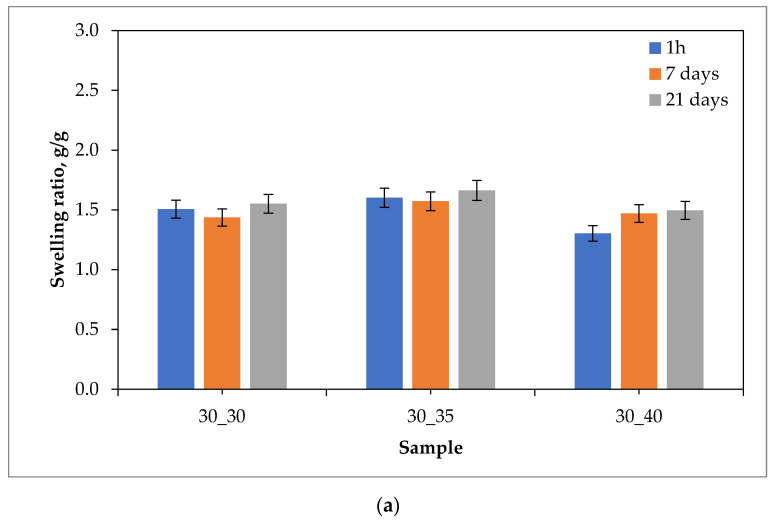

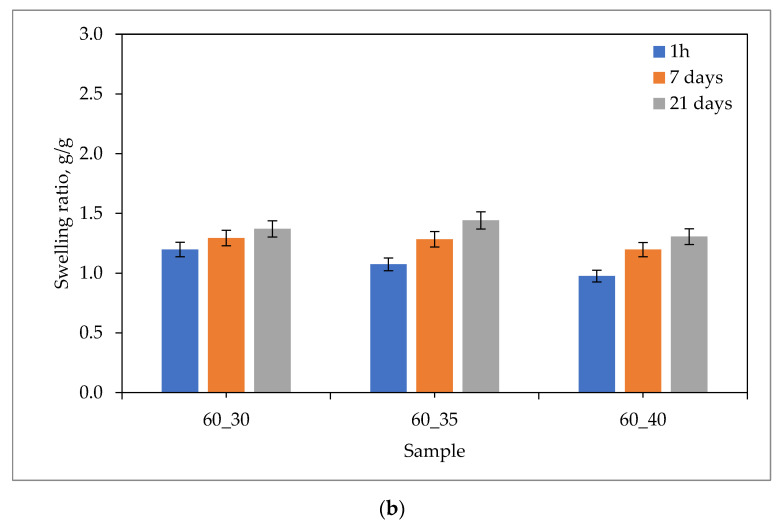
Results of swelling studies of hydrogels containing 2.0 g (**a**) and 4.0 g (**b**) of royal jelly in artificial saliva.

Results of the swelling studies in the form of bar charts showing the values of the swelling ratios (as an average value from three measurements (n = 3) ± standard deviation (SD)) calculated for each hydrogel sample are presented below in Figure 1, Figure 2, Figure 3 and Figure 4.

The analysis of the results of the performed investigations allowed to us observe the typical for PVP-based hydrogel materials tendency to decrease the value of the swelling ratio with an increasing amount of the crosslinking agent used during the photopolymerization process. The reason for this dependence is the increase in the crosslinking degree of the material for which the above-mentioned crosslinking agent is responsible, which at the synthesis stage builds into the structure of polymer chains to form a three-dimensional spatial structure. This process is called bridging. Thus, materials containing a larger amount of crosslinking agent are characterized by a more packed structure of polymer networks, which prevents the solution from being absorbed as much as in the case of materials containing a smaller amount of this reactant. The above-described dependence may be attributed to most of the materials obtained, except for those tested in distilled water. The reason for this may also be the crosslinking density of the hydrogel matrix, which is affected not only by the amount of crosslinking agent, but also by the presence of ions from the incubation fluids. Ringer liquid, SBF, and artificial saliva all contain ions, including divalent Ca^2+^ ions, which may build into the structure of the polymer chains, further thickening it by making it more difficult for the incubation fluid to penetrate the hydrogel material. Distilled water, as it is devoid of the aforementioned ions, does not affect the cross-linking density of the structure and thus the swelling ratio.

The vast majority of the materials tested were characterized by a gradual increase in the swelling ratio with the time at which the study was performed. This means that the materials had most likely not yet absorbed the maximum amount of fluids after 21 days. However, it may be noticed that in the case of some samples, a reduced value of the swelling ratio after 21 days of swelling compared to the value calculated after 7 days of swelling was observed. This may indicate the partial degradation of these materials or the release of the modifier (the royal jelly) from their interior.

The highest sorption capacity was demonstrated for materials tested in distilled water. This is due to the absence of ions in this environment, which may build into the structure of polymer chains and affect the absorption of fluid by the material. On the other hand, the lowest swelling ratio was observed for materials tested in artificial saliva, as it contains the most divalent Ca^2+^ ions, which negatively affect the sorption capacity of hydrogels.

Importantly, a clear difference between the sorption properties of the materials containing 2 g of royal jelly (30_30, 30_35, 30_40) and those with 4 g of royal jelly (60_30, 60_35, 60_40) may also be noted. Hydrogels with twice the amount of royal jelly show worse sorption properties. This is most likely due to the depositing of more of this additive in the spaces between the polymer chains, which reduces the ability to absorb as much liquid as materials with less content of royal jelly. This also shows the inclusion of the additive in the hydrogel matrix, which is a decent effect.

The analysis of the results also makes it possible to observe that the difference in the value of the swelling index between measurements after 1 h, 7 days and 21 days was greater for materials containing 4 g of royal jelly than for materials containing 2 g of this additive. Most likely, materials with more royal jelly also release more of this additive. More spaces between the polymer chains are then released, which consequently leads to the absorption of more liquid.

Investigations showing a similar effect of royal jelly on the swelling properties of hydrogel materials were also performed in [35,36,37,38,39,40].

### 2.2. Characterization of Hydrogels in the Environment of Simulated Body Fluids

**Figure 5:**
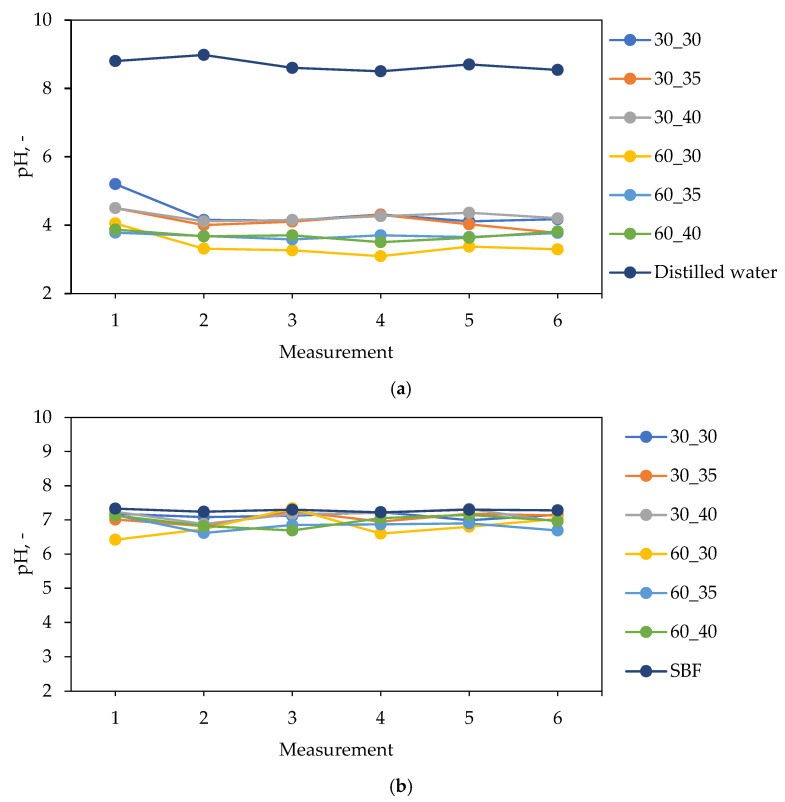
Results of incubation studies of hydrogels in (**a**) distilled water and (**b**) SBF.

**Figure 6:**
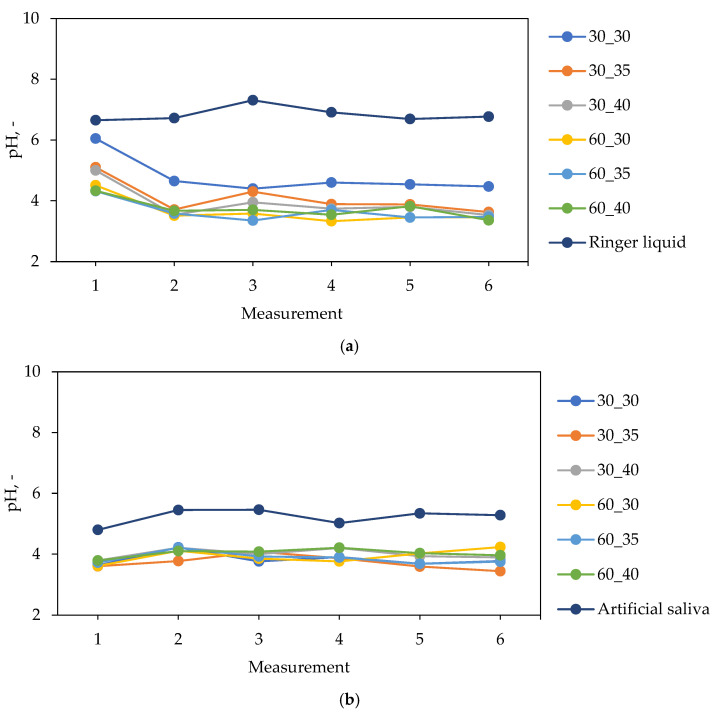
Results of incubation studies of hydrogels in (**a**) Ringer liquid and (**b**) artificial saliva.

The graphs showing the changes in pH values of simulated physiological liquids during the incubation of hydrogel samples are presented in Figure 5 and Figure 6.

In Figure 5 and Figure 6, small differences in the pH values of all tested liquids can be observed. This indicate that the hydrogels show stability in all environments. Additionally, the lack of spikes in pH values indicates that the tested samples did not degrade over the time in which the test was conducted.

Materials incubated in distilled water significantly reduced their pH from a value of about 8.5 to a value of about 4.0. A major reason affecting the lowering of the pH value is the release of royal jelly, which contains about 5% organic acids, mainly pyruvic acid and lactic acid, to which it owes a pH in the range of 3.4–4.3. Such large changes in pH are most likely due to the lack of divalent Ca^2+^ ions, which could have resulted in an increase in the cross-linking density of the material, which would consequently lead to less release of the modifying substance. Materials incubated in artificial saliva and Ringer liquid also lowered the pH of the incubated fluids, but not as much as in the case of distilled water. This is due to the presence of ions that, by building into the structure of the polymer chains, limit the release of the modifying substance. In the case of materials incubated in SBF, the pH changes were the smallest among the fluids used. The reason for this is most likely the ion-rich composition of SBF, which contributes to increasing the crosslinking degree of the hydrogels, and this results in a negligible release of royal jelly compared to the incubation in the other fluids.

Slight differences between materials with different royal jelly contents can also be observed. Hydrogel samples 60_30, 60_35, and 60_40 lowered the pH of the incubated fluids slightly more than materials 30_30, 30_35, and 30_60 because they contain twice as much royal jelly, which has an acidic pH.

A discussion on the behavior of the hydrogels containing royal in simulated body fluids was also performed in [41,42,43,44,45].

### 2.3. Evaluation of Hydrogel Surface Morphology Using SEM Microscopy

**Figure 7:**
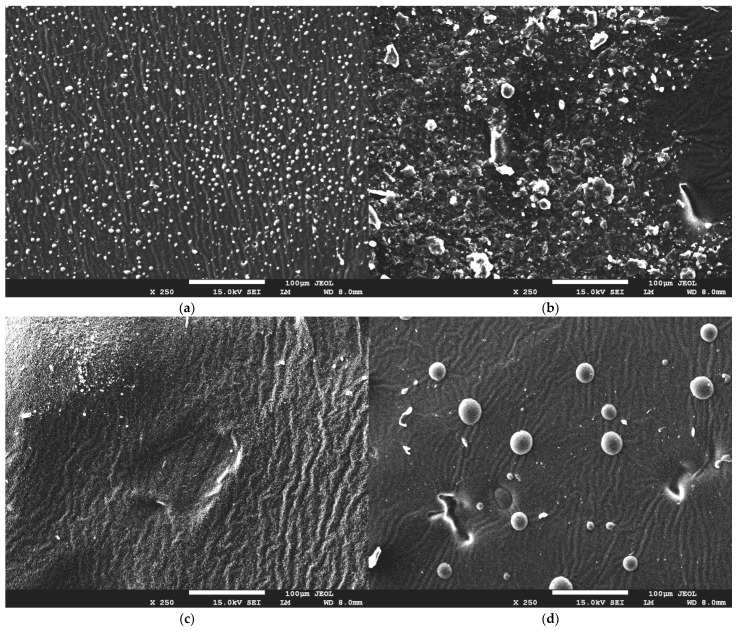
SEM images of hydrogel materials containing different contents of crosslinking agent and royal jelly: (**a**) sample 30_30, (**b**) sample 30_40, (**c**) sample 60_30, and (**d**) sample 60_40.

In Figure 7, SEM images of the hydrogels are presented.

SEM micrographs show us that the addition of royal jelly to hydrogel materials manifests in the appearance of visible granules on the surface, with a spherical, regular shape. A higher amount of crosslinking agent causes an intense breakout of granules and irregularities on the surface of the hydrogel material. This is related to the polymerization process, in which appropriate proportions of components during the polymerization reaction lead to a material with a more uniform structure or a material with visible morphological changes. Nevertheless, the structure of the obtained materials is undulating and porous, indicating that there is no effect of bee milk on the hydrogel structure itself.

### 2.4. Wettability of Hydrogel Materials

The results of the experiments showing the wetting angles both for distilled water and diiodomethane, as well as the values of the polar surface energy and the dispersion energy, respectively, have been presented in Table 1. Contact angle measurements were performed three times for each hydrogel sample.

The presented results show the contact angle and surface free energy measurements for four different samples, while the samples were tested using two measuring liquids, i.e., distilled water and diiodomethane. Additionally, the surface free energy values were divided into dispersive and polar components, and the total free energy was calculated.

Contact angle measurements for distilled water indicate that samples 30_30 and 30_40 have higher contact angles than samples 60_30 and 60_40. This suggests that samples 30_30 and 30_40 have lower hydrophilicity and higher hydrophobicity than samples 60_30 and 60_40. The contact angle measurements for diiodomethane show that sample 30_40 has the lowest contact angle, indicating the highest hydrophilicity among all samples. Samples 30_30 and 60_40 show similar contact angle values, indicating similar hydrophilic properties, while sample 60_30 has the highest contact angle, indicating the lowest hydrophilicity.

Surface free energy measurements show that samples 30_40 and 60_40 have the highest dispersive surface free energy values, while samples 30_30 and 60_30 have lower values. This suggests that samples 30_40 and 60_40 have higher levels of non-polar interactions, which could be attributed to the presence of hydrophobic groups in the sample. On the other hand, polar surface free energy values are significantly lower for all samples, with sample 30_40 having the highest value, suggesting that polar interactions play a minor role in the sample’s surface properties. The total free energy values follow the same trend as the dispersive surface free energy values, where samples 30_40 and 60_40 have the highest values, and samples 30_30 and 60_30 have lower values.

Overall, the results suggest that samples 30_30 and 60_30 have lower hydrophilicity and higher hydrophobicity compared to samples 30_40 and 60_40. The lower polar surface free energy values suggest that polar interactions play a minor role in the surface properties of all samples. However, samples 30_40 and 60_40 have higher dispersive surface free energy values, indicating a higher level of non-polar interactions, which could be attributed to the presence of hydrophobic groups. These findings could have implications for the material’s properties, such as wettability, adhesion, and surface roughness, and should be taken into consideration when selecting materials for specific applications. Similar conclusions were drawn also in other works [46,47].

### 2.5. Study of Mechanical Properties of Hydrogel Materials

**Figure 8:**
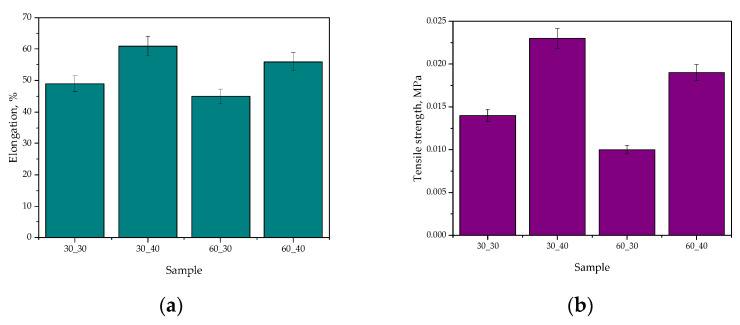
Results of the mechanical studies involving the determined percentage elongation (**a**) and the tensile strength (**b**) of tested hydrogels.

In Figure 8, the results of the mechanical studies, including both the tensile strength and the percentage elongation, are presented (as an average value from three measurements (n = 3) ± standard deviation (SD)).

The present study verified the mechanical properties of hydrogel materials containing royal jelly. The hydrogels were prepared using polyvinylpyrrolidone (PVP) as the main component of the polymer matrix, various contents of royal jelly, various contents of cross-linking agent, and photoinitiator. The samples were characterized for their tensile strength and elongation percentage. The results showed that the addition of bee milk increased the elongation percentage while decreasing the tensile strength of the hydrogels.

Similar studies were performed by Ahmed et al. [48]. They verified the effect of honey on the mechanical properties of chitosan-based hydrogels. The results showed that the addition of honey increased the elongation percentage while decreasing the tensile strength of the hydrogels. The authors explained this effect with the presence of hydrogen bonding between the honey and chitosan molecules, which enhanced the elasticity and flexibility of the hydrogels.

Comparing the results of the present study with those of Ahmed et al., it is evident that the addition of natural additives to hydrogels may significantly impact their mechanical properties. In these studies, the addition of natural additives increased the elongation percentage while decreasing the tensile strength of the hydrogels. These findings suggest that natural additives can be used to modify the mechanical properties of hydrogels for specific applications that require high flexibility and elasticity.

In conclusion, the present study investigated the effect of royal jelly on the mechanical properties of hydrogels. The results showed that the addition of royal jelly increased the elongation percentage while decreasing the tensile strength of the hydrogels. These findings are consistent with previous studies that have investigated the effects of natural additives on the mechanical properties of hydrogels. The results suggest that natural additives can be used to modify the mechanical properties of hydrogels for specific applications.

**Figure 9:**
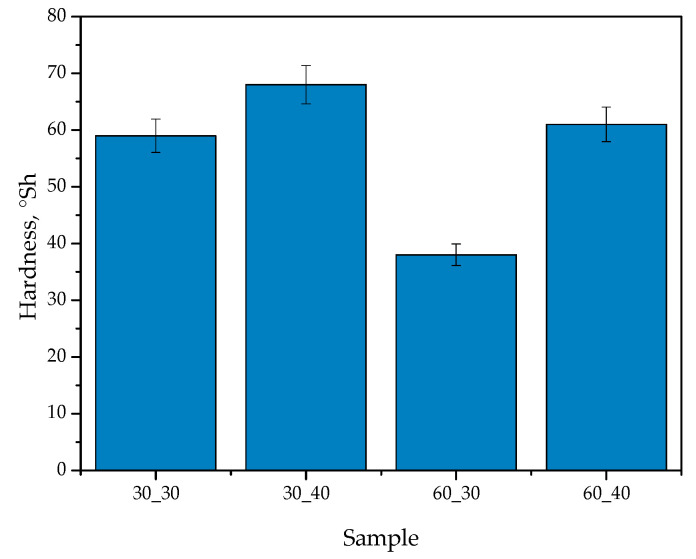
Hardness of hydrogel materials.

In Figure 9, the results of the analysis of hydrogels’ hardness (as an average value from five measurements (n = 5) ± standard deviation (SD)) are presented.

The results of the Shore A hardness test showed that the hardness of hydrogels increased with an increase in the concentration of cross-linker. The sample with the highest concentration of cross-linker (sample 30_40) had the highest hardness value of 68°Sh, while the sample with the lowest concentration (sample 60_30) had the lowest hardness value of 38°Sh. This trend can be attributed to the formation of a denser network of polymer chains, resulting in a more rigid structure.

The addition of royal jelly to the hydrogel also affected the hardness of the samples. The sample with the highest concentration of royal jelly (sample 60_40) had a higher hardness value of 61°Sh compared to the sample with a lower concentration of royal jelly (sample 30_30), which had a hardness value of 59°Sh. This can be explained by the fact that royal jelly contains a high concentration of proteins and enzymes that can act as cross-linkers, resulting in a more compact structure.

The results of this study are consistent with previous studies that have investigated the effect of cross-linking agents on the hardness of hydrogels. For instance, Zhao et al. [49] found that the hardness of chitosan hydrogels increased with an increase in the concentration of the cross-linking agent. Similarly, Ren et al. [50] reported that the hardness of polyvinyl alcohol (PVA) hydrogels increased with an increase in the concentration of glutaraldehyde, which is a common cross-linking agent.

### 2.6. Imaging of Hydrogels by Means of Optical Microscopy

**Figure 10:**
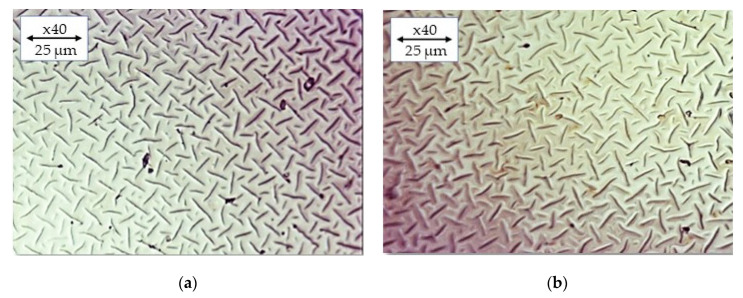

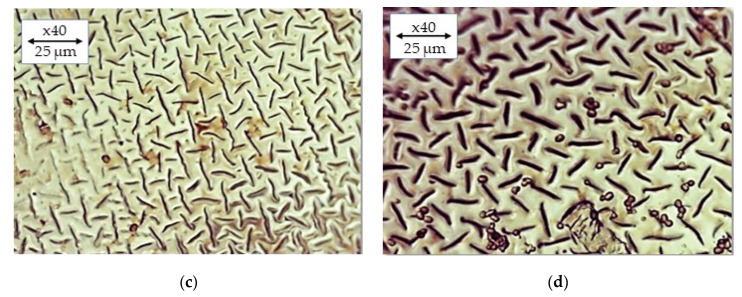
Optical microscope images of hydrogel materials containing different contents of crosslinking agent and royal jelly: (**a**) sample 30_30, (**b**) sample 30_40, (**c**) sample 60_30, and (**d**) sample 60_40).

In Figure 10, the optical microscope images for the selected hydrogel materials are presented.

The optical microscopy analysis of PVP-based hydrogels modified with royal jelly revealed a smooth and uniform surface morphology. No significant differences in surface morphology were observed between the samples with varying compositions of crosslinking agents or royal jelly content. These results suggest that the addition of royal jelly does not affect the surface morphology of PVP-based hydrogels. Similar findings were reported by Li et al. in their study on the effect of silver nanoparticles on the morphology of PVP hydrogels. The authors found that the addition of silver nanoparticles did not significantly alter the surface morphology of the hydrogels. In another study by Wang et al. investigating the effect of chitosan content on the surface morphology of hydrogels, the authors reported no significant changes in surface morphology with increasing chitosan concentration [51,52].

Overall, these results suggest that the surface morphology of hydrogels is not significantly affected by the addition of various components, including crosslinking agents and bioactive compounds such as royal jelly and silver nanoparticles.

### 2.7. Results of the FT-IR Spectroscopy

**Figure 11:**
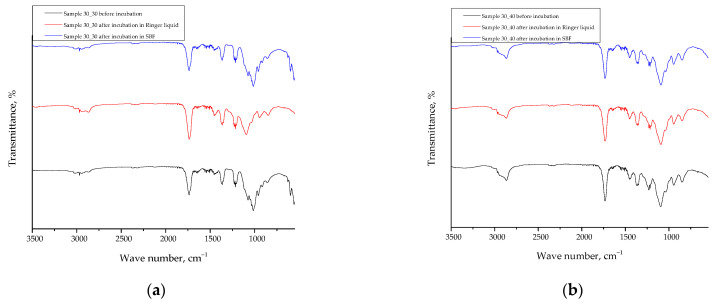

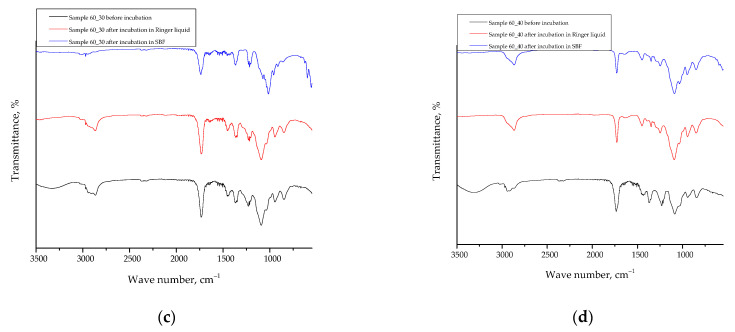
FT-IR spectra of hydrogels (**a**) sample 30_30, (**b**) sample 30_40, (**c**) sample 60_30, and (**d**) sample 60_40 before and after incubation in selected simulated physiological liquid.

Below, in Figure 11, the FT-IR spectra of tested hydrogels are presented, while in Table 2, a summary of the absorption bands visible on FT-IR spectra with their assignment to the appropriate groups present within the structure of the reagents applied has been compiled.

The FT-IR analysis of the hydrogel materials before and after incubation in Ringer’s solution and SBF showed no significant differences, indicating the absence of degradation during the incubation period. The hydrogel materials studied were PVP-based polymeric hydrogels modified with royal jelly. The absence of any changes in the FT-IR spectra suggests that the structure of the hydrogel materials remained intact after incubation in the test solutions.

Similar results were reported by Chen et al. [59] in their study on the stability of hydrogels based on sodium alginate and chitosan after immersion in simulated body fluid. The authors found no significant changes in the FT-IR spectra of the hydrogels before and after immersion, indicating the stability of the hydrogel structure.

In another study by Kim et al. [60] investigating the stability of hydrogels based on hyaluronic acid and gelatin after immersion in phosphate-buffered saline, the authors also reported no significant changes in the FT-IR spectra of the hydrogels. This further supports the stability of hydrogel materials in physiological environments.

## 3. Materials and Methods

### 3.1. Materials

Polyvinylpyrrolidone (PVP, average molecular weight 58,000 g/mol), poly(ethylene glycol) diacrylate (PEGDA 700—average molecular weight 700 g/mol), and 2-hydroxy-2-methylpropiophenone (97%) were purchased from Sigma Aldrich (Saint Louis, MO, USA). Royal jelly, on the other hand, was purchased from apini.pl (Ziółkowski Juventas S.K.A., Jurowce, Poland). All reagents were used as received without further purification.

### 3.2. Synthesis of Hydrogel Dressings under the Influence of UV Radiation

The photopolymerization process using UV radiation was chosen as the method for obtaining hydrogels. This was selected considering the short reaction time, low energy requirements, and lack of formation of by-products, as well as the possibility of simultaneous sterilization of the resulting materials by UV radiation, which is particularly important due to the potential application of these materials for biomedical purposes. Polyvinylpyrrolidone (PVP) was selected as the main component of the hydrogel matrix while the royal jelly was applied as a modifier. In turn, poly(ethylene glycol) diacrylate, with an average molecular weight of 700 g/mol, was employed as a crosslinking agent, and 2-hydroxy-2-methylpropiophenone was used to initiate the photopolymerization process.

**Figure 12:**
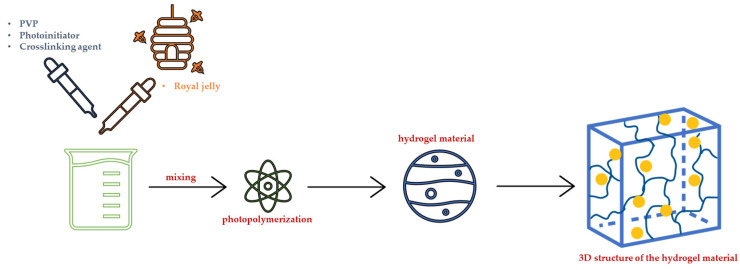
The scheme of the synthesis of hydrogel materials.

In order to obtain hydrogels, appropriate amounts of all reactants were mixed and exposed to UV radiation. Photopolymerization was carried out for 120 s using an EMITA VP-60 lamp (power 180 W, λ = 320 nm, manufactured by Famed, Lodz, Poland) as the radiation source. The synthesis of hydrogels is schematically presented in Figure 12.

Table 3 presents the detailed composition of the obtained hydrogels.

**Figure 13:**
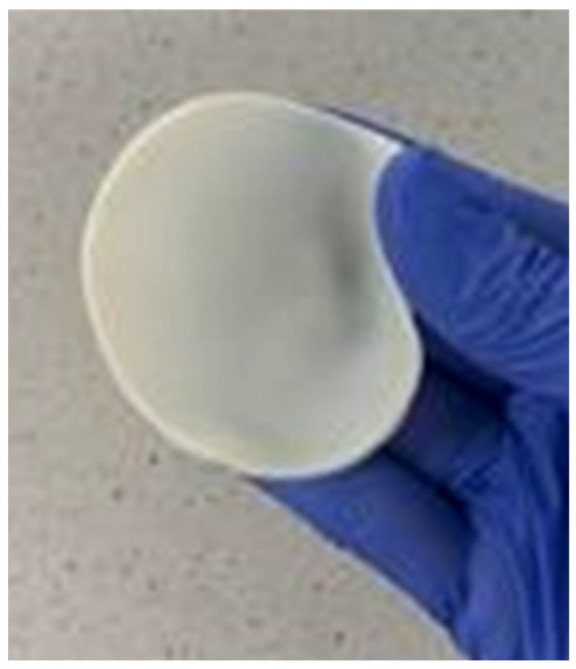
The exemplary hydrogel sample (sample 30_30).

In Figure 13, an exemplary image of the obtained hydrogel is presented.

Next, selected physicochemical properties of the hydrogels were verified and discussed, focusing primarily on the effect of the average molecular weight of the crosslinking agent and content of the royal jelly in the hydrogel matrix.

### 3.3. Sorption Capacity of Hydrogel Dressings

The swelling ability of dressing materials allows them to absorb various solutions in contact with these materials. Hence, the dressing materials with this function may absorb wound exudate. This, in turn, may speed up the wound-healing process. Therefore, the developed materials were tested precisely for this property.

Swelling properties of hydrogels were verified with the use of simulated physiological fluids, i.e., Ringer liquid (balanced isotonic crystalloid fluid containing physiological concentrations of the following ions: Ca^2+^, K^+^, Na^+^, and Cl^−^ [61]), simulated body fluid (SBF, isotonic to human blood plasma [62]), artificial saliva, and distilled water (as a reference liquid). Firstly, hydrogel samples weighing 1.0 g were dried for 24 h at 37 °C, and introduced into 50 mL of the aforementioned fluids. After 1 h, the swollen samples were separated from the liquids, weighed again and re-introduced into the absorbed medium. The procedure was repeated after 7 and 21 days. The sorption capacity of the hydrogels was determined by the swelling ratio (α), calculated using the following Equation (1):

(1) $$\alpha =\frac{\left(m_{p}-m_{s}\right)}{m_{p}}$$

where: *α*—swelling ratio, g/g; $m_{s}$ —mass of swollen hydrogel sample, g; $m_{p}$ —initial mass of hydrogel sample (before swelling), g.

The study was performed in triplicate for each hydrogel sample.

### 3.4. Characterization of Hydrogels in the Environment of Simulated Physiological Body Fluids

In this study, hydrogel samples (weighing approximately 1.0 g) were placed in 50 mL of selected physiological fluids for 18 days. Incubation was carried out in the same liquids as the sorption investigations, using SBF, Ringer liquid, artificial saliva, and distilled water. The study was conducted at 37 °C, thus simulating the temperature conditions of the human body. During the incubation period, the pH and the temperature of the incubation fluids were measured using the ELMETRON CX-701 multifunctional meter (Elmetron, Zabrze, Poland).

### 3.5. Evaluation of Hydrogel Surface Morphology Using SEM Technique

The highly dried hydrogel materials were investigated using the JEOL JSM-7500F scanning electron microscope (Jeol Ltd., Tokyo, Japan). The study aimed at verifying the impact of the hydrogel compositions on their surface morphology. Prior to testing, hydrogels directly after the synthesis were dried at 37 °C for 24 h. Then, the samples with dimensions 1.0 cm × 1.0 cm × 0.1 cm were cut from the hydrogels and sputtered with chromium to provide a layer on the hydrogels’ surfaces. All microscopic analyses were carried out at room temperature.

### 3.6. Wettability of Hydrogel Materials

In order to determine the hydrophilicity of the hydrogels, the wetting angles of their surfaces were verified. In general, the wetting angles were determined by droplet shape analysis performed using the Kruss DSA 100M (A.KRÜSS Optronic GmbH, Hamburg, Germany) apparatus and consisted of placing the hydrogel sample on a stationary table and then dropping the measuring liquid (distilled water) with a micropipette onto the tested sample. The images of the drop shapes formed in contact with the analyzed hydrogels were recorded via the optical system using a digital camera. Additionally, the surface free energies of hydrogels were also calculated, while the detailed method of their determining has been thoroughly described in our previous paper [63]. The analysis was performed in triplicate for each hydrogel sample.

### 3.7. Characterization of the Mechanical Properties of Hydrogel Materials

The mechanical properties of the hydrogels, including their tensile strength, percentage elongation, and hardness, were also characterized. The paddle-shaped hydrogel samples were prepared using a ZCP020 (ZwickRoell GmbH & Co. KG, Ulm, Germany) manual die-cutting press, and then dried under the constant load (to maintain a shape) at 37 °C for 5 days. Next, the samples were analyzed using the Brookfield CT3 Texture Analyzer (AMETEK Brookfield, Middleboro, MA, USA). The tests consisted of placing the analyzed sample between the jaws of the testing machine and moving the jaws of the machine away from each other until the sample broke. The procedure allowed us to determine the tensile strength of the hydrogels (*R_m_*) and their percentage elongation (*A*), which were calculated using the following Equations (2) and (3):

(2) $$R_{m}=\frac{F_{m}}{S_{0}}$$

(3) $$A=\frac{100\times (I_{u}-I_{0})}{I_{0}}$$

where: $R_{m}$ —tensile strength, $F_{m}$ —the maximum strength, $S_{0}$ —the cross-sectional area of the analyzed sample before the analysis, A —the percentage elongation, $I_{u}$ —the measuring length after the sample was ruptured, and $I_{0}$ —the measuring length of the sample before the measurement. Studies were performed in triplicate for each hydrogel sample.

Studies included also the evaluation of the hardness of hydrogels. To determine this property, the Insize Shore A Hardness tester (Insize Inc., Loganville, GA, USA) was employed. The hydrogel samples were pressed against the tester’s base while a needle-like indenter hammered into it. Once a balance between the material and the pressure was established, the tester provided the appropriate hardness value. For each sample, five measurements were performed. The analysis was conducted at room temperature.

### 3.8. Analysis of the Impact of the Incubation in Simulated Physiological Liquids on the Structure of Hydrogel Materials via Fourier Transform Infrared (FT-IR) Spectroscopy

FT-IR spectroscopy was used to analyze the effect of simulated physiological liquids on the chemical structure of hydrogels. The method involves comparing absorption bands of functional groups present in the samples before and after incubation. The study was performed at room temperature using a Thermo Scientific Nicolet iS5 (Thermo Fisher Scientific, Waltham, MA, USA) FT-IR spectrophotometer, and the spectra were recorded in the range of 4000–500 cm^−1^ with a resolution of 4.0 cm^−1^. The hydrogel samples before the analysis were dried at 37 °C for 24 h.

### 3.9. Analysis of the Hydrogels via the Optical Microscope

The hydrogel sample (dried previously at 37 °C for 24 h) was placed on a microscope slide and covered with a cover slip. It was then ensured that the sample was evenly distributed on the microscope slide and covered with a cover slip without any air bubbles. Images were recorded using the Delta Optical Genetic Bino microscope (Delta Optical, Warsaw, Poland). The study was performed at room temperature.

## 4. Conclusions

Based on the research, it may be concluded that the hydrogels showed swelling ability in simulated physiological liquids wherein the highest swelling capacity was reported in distilled water, and the lowest one in artificial saliva. This was strongly related to the composition of the tested media—ions present in the absorbed liquid increase the crosslinking density of the materials, thus lowering their sorption capacity. The investigated materials also demonstrated stability in simulated physiological liquids—any changes in their structure resulted from the incubation were not observed, which was verified via FT-IR spectroscopy. The differences in pH of incubation liquids with hydrogels compared to reference liquids probably resulted from the release of the royal jelly from the materials. All analyzed hydrogels showed a relatively homogeneous surface wherein any dependence between the hydrogel composition (royal jelly or crosslinking agent content) and the surface morphology was not observed. The content of royal jelly affected, in turn, the wettability of hydrogels—the higher its content, the higher contact angle value. This modifier also affected the mechanical properties of hydrogels—the higher royal jelly content, the higher the percentage of elongation and hardness and the lower the tensile strength. Importantly, the higher the crosslinker content in hydrogels, the higher their hardness. Thus, it was proven that the modification of hydrogels with royal jelly resulted in the preparation of materials showing promising properties in terms of their potential application as dressing materials. To fully verify the usefulness of the developed materials it is necessary to determine, among other things, their cytotoxicity and proinflammatory activity, as well as the possible release of the modifier from their polymer network.

## Figures and Tables

**Table 1:** The values of wetting angles of hydrogels both for distilled water and diiodomethane and the calculated surface energies for these angles (shown as an average value from three measurements (n = 3) ± standard deviation (SD)).

Sample	Contact Angle, °		Surface Free Energy		
	Distilled Water	Diiodomethane	Polar, mJ/m^2^	Dispersive, mJ/m^2^	Total Free Energy, mJ/m^2^
30_30	113 ± 2.0%	48 ± 0.8%	1.72	45.73	47.44
30_40	122 ± 2.4%	35 ± 1.2%	6.75	58.44	65.19
60_30	94 ± 2.8%	56 ± 1.7%	0.90	33.69	34.59
60_40	105 ± 1.8%	51 ± 1.1%	0.16	40.87	41.03

**Table 2:** The absorption bands visible on the FT-IR spectra with assigned functional groups derived from the reagents applied during the synthesis of hydrogels [53,54,55,56,57,58].

Wave Number, cm^−1^	Functional Group	Substrate
2930	-C-H	PVP
1650	-C=O	
1280	-C-N	
2925	-C-H	Crosslinking agent
1735	-C=O	
1640	-C=C	
3000	-C-H	Photoinitiator
1715	-C=O	
1600	-C=C	
3000–2800	-CH and -CH_2_	Royal yelly
1700–1600	-C=O	
1500–1300	-OH and -CH_2_-CH_2_-	
1200–900	-C-O and -C-C-	

**Table 3:** Composition of the obtained hydrogel materials.

Sample	15% PVP Solution, mL	Royal Jelly, wt.% *	Photoinitiator, mL	Crosslinking Agent, *v*/*v* % **
30_30	7	30	0.05	30
30_35				35
30_40				40
60_30		60		30
60_35				35
60_40				40

* relative to PVP content. ** relative to PVP content.

## Data Availability

Not applicable.
