# Correction: Kudłacik-Kramarczyk et al. Exploring the Potential of Royal-Jelly-Incorporated Hydrogel Dressings as Innovative Wound Care Materials. *Int. J. Mol. Sci.* 2023, *24*, 8738

**DOI:** 10.3390/ijms25084280

**Published:** 2024-04-12

**Authors:** Sonia Kudłacik-Kramarczyk, Marcel Krzan, Mateusz Jamroży, Alicja Przybyłowicz, Anna Drabczyk

**Affiliations:** 1 Department of Materials Engineering, Faculty of Materials Engineering and Physics, Cracow University of Technology, 37 Jana Pawła II Av., 31-864 Krakow, Poland; mateusz.jamrozy@student.pk.edu.pl (M.J.); alicja.przybylowicz@student.pk.edu.pl (A.P.); anna.drabczyk2@pk.edu.pl (A.D.); 2 Jerzy Haber Institute of Catalysis and Surface Chemistry, Polish Academy of Sciences, 8 Niezapominajek St., 30-239 Krakow, Poland

The authors wish to make the following corrections to this paper [1]: In the original publication, there were mistakes in Figure 7 and the main text as published. The original figure is not of the right sample, which led to the wrong discussion in Section 2.3. We have re-imaged these materials and included the corrected images in this figure. The corrected Figure 7 and the main text appear below.

Section 2.3 on page 9. The second paragraph is changed to the following: SEM micrographs show us that the addition of royal jelly to hydrogel materials manifests in the appearance of visible granules on the surface, with a spherical, regular shape. A higher amount of crosslinking agent causes an intense breakout of granules and irregularities on the surface of the hydrogel material. This is related to the polymerization process, in which appropriate proportions of components during the polymerization reaction lead to a material with a more uniform structure or a material with visible morphological changes. Nevertheless, the structure of the obtained materials is undulating and porous, indicating that there is no effect of bee milk on the hydrogel structure itself.

Section 3.5 on page 17. Line 1, the sentence is changed to the following: The highly dried hydrogel materials were investigated using the JEOL JSM-7500F scanning electron microscope (Jeol Ltd., Tokyo, Japan). Line 4–6, the sentence is changed to the following: Then, the samples with dimensions 1.0 cm × 1.0 cm × 0.1 cm were cut from the hydrogels and sputtered with chromium to provide a layer on the hydrogels’ surfaces.

The authors state that the scientific conclusions are unaffected. This correction was approved by the Academic Editor. The original publication has also been updated.

## Figures and Tables

**Figure 7:**
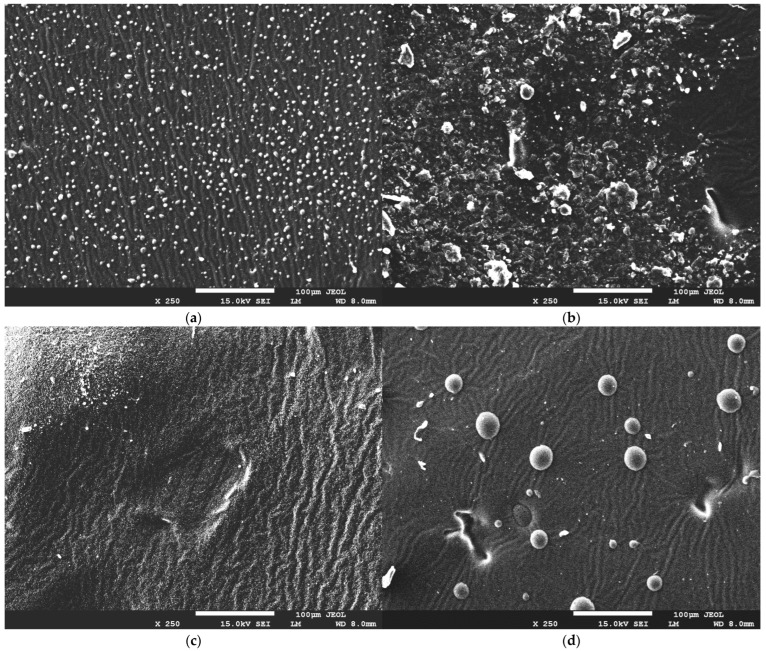
SEM images of hydrogel materials containing different contents of crosslinking agent and royal jelly: (**a**) sample 30_30, (**b**) sample 30_40, (**c**) sample 60_30, and (**d**) sample 60_40.
